# A haploproficient interaction of the transaldolase paralogue *NQM1* with the transcription factor *VHR1* affects stationary phase survival and oxidative stress resistance

**DOI:** 10.1186/s12863-015-0171-6

**Published:** 2015-02-11

**Authors:** Steve Michel, Markus A Keller, Mirjam MC Wamelink, Markus Ralser

**Affiliations:** Max Planck Institute for Molecular Genetics, Ihnestr 73, Berlin, 14195 Germany; Department of Biochemistry and Cambridge Systems Biology Center, University of Cambridge, 80, Tennis, Court Road, Cambridge, CB2 1GA UK; MRC National Institute for Medical Research, The Ridgeway, Mill Hill, London, UK; Metabolic Unit, Department of Clinical Chemistry, VU University Medical Centre Amsterdam, Amsterdam, The Netherlands

**Keywords:** Chronological lifespan, Pentose phosphate pathway, Transaldolase, NQM1, VHR1, Oxidative stress

## Abstract

**Background:**

Studying the survival of yeast in stationary phase, known as chronological lifespan, led to the identification of molecular ageing factors conserved from yeast to higher organisms. To identify functional interactions among yeast chronological ageing genes, we conducted a haploproficiency screen on the basis of previously identified long-living mutants. For this, we created a library of heterozygous *Saccharomyces cerevisiae* double deletion strains and aged them in a competitive manner.

**Results:**

Stationary phase survival was prolonged in a double heterozygous mutant of the metabolic enzyme *non-quiescent mutant 1 (NQM1*), a paralogue to the pentose phosphate pathway enzyme transaldolase (*TAL1*), and the transcription factor *vitamin H response transcription factor* 1 (*VHR1*). We find that cells deleted for the two genes possess increased clonogenicity at late stages of stationary phase survival, but find no indication that the mutations delay initial mortality upon reaching stationary phase, canonically defined as an extension of chronological lifespan. We show that both genes influence the concentration of metabolites of glycolysis and the pentose phosphate pathway, central metabolic players in the ageing process, and affect osmolality of growth media in stationary phase cultures. Moreover, *NQM1* is glucose repressed and induced in a *VHR1* dependent manner upon caloric restriction, on non-fermentable carbon sources, as well as under osmotic and oxidative stress. Finally, deletion of *NQM1* is shown to confer resistance to oxidizing substances.

**Conclusions:**

The transaldolase paralogue *NQM1* and the transcription factor *VHR1* interact haploproficiently and affect yeast stationary phase survival. The glucose repressed *NQM1* gene is induced under various stress conditions, affects stress resistance and this process is dependent on *VHR1*. While *NQM1* appears not to function in the pentose phosphate pathway, the interplay of *NQM1* with *VHR1* influences the yeast metabolic homeostasis and stress tolerance during stationary phase, processes associated with yeast ageing.

**Electronic supplementary material:**

The online version of this article (doi:10.1186/s12863-015-0171-6) contains supplementary material, which is available to authorized users.

## Background

Eukaryotic cells possess a variety of different strategies to maintain homeostasis, and adjust metabolism upon changes in environment and to survive stress situations such as oxido-reductive stress, osmotic turbulences, exposure to toxins, temperature imbalances or fluctuating nutrient concentrations [1-5]. This cellular flexibility is a requirement for a successful ageing process. It has thus become common view that cellular stress response mechanisms are key players in gerontology. Transcriptional regulation is important in cellular adaptation to both stress and ageing, and many stress responsive genes and regulatory systems have been identified [6-9]. The transcriptional response varies between stress conditions, but many gene sets and control mechanisms overlap and play a role in multiple situations. Key proteins required to establish the transcriptional stress response include the transcription factors Yap1p, Msn2/4p, Hsf1p, Hog1p/Hot1p [10-12]. These transcriptional regulators allow the cell to react directly to the applied stress condition and induce effective defense mechanisms such as molecular chaperones and anti-oxidant proteins. However, transcriptional regulation does not explain the full spectra of cellular adaptations to changing environments and nutrition [13-15]. A presumably equally important, complementary strategy to react to environmental turbulences is to adjust cellular metabolism. A challenge for the cell in stress situations is to maintain the canonical function of metabolism, which is to continuously provide metabolites and cofactors such as ATP and NAD(P)H, required for the cell to survive and to continue proliferation upon the end of the stress situation. In particular, the ancient metabolic routes of glycolysis and the pentose phosphate pathway, amino acid and polyamine synthesis are at the centre of cellular metabolism, and have been shown to be involved in establishing fast and effective strategies in responding to situations like oxidative stress [4,5,16-18].

Genome duplication was one of the triggers that facilitated evolutionary adaptations to improve functionality of metabolism in stress situations. In budding yeast, a whole genome duplication event has been dated approximately 100 million years ago [19]. This created the basis for the evolution of isozymes that obtained specific functions during the stress response [20,21]. A diversification of enzyme function in stress situations has been reported for isozymes of isocitrate dehydrogenases [22,23], alcohol dehydrogenases [24], sirtuins [25,26], nutrient transporters [27] and glycolytic enzymes. The latter includes the most downstream glycolytic enzyme pyruvate kinase (isozymes *PYK1* and *PYK2*), where the low-activity orthologue *PYK2* is induced under conditions of high respiratory activity and protects the cells from oxidative damage [4].

Here we present evidence that *NQM1* [28]*,* a paralogue of the pentose phosphate pathway (PPP) enzyme transaldolase *TAL1*, as well as its regulating transcription factor *VHR1* [29] are required during osmotic and oxidative stress, and affect survival of yeast cells upon long time starvation. We identified a heterozygous deletion of both genes through enrichment in a haploproficiency screen targeting genes previously implicated in chronological lifespan [30]. In subsequent analysis we find that *NQM1* is induced upon nutrient limiting-, osmotic- and oxidative stress conditions and that this induction is dependent on the presence of *VHR1*. Finally, we show that the two genes cooperate in achieving oxidative stress tolerance of wild type cells.

## Methods

### Yeast strains and cultivation

All yeast strains used in this study are derived from the parent strain S288C. Wild type reference strains are haploid BY4741 (*MAT* a) or diploid BY4743 (*MAT* a/ *MAT* α) and were derived from the international knock out collection [31] obtained from EUROSCARF (Frankfurt, Germany; a complete list of deletion mutants used in this study is given as Additional file 1: Table S1).

Yeast was cultivated as detailed previously [32] at 30°C either in liquid or on solid (agar) yeast-extract peptone dextrose complex media (YPD: 20 g/l peptone, 10 g/l yeast extract, 20 g/l agar, 2% glucose) or in synthetic complete media (SC: 6,8 g/l yeast nitrogen base with ammonium sulfate, 0.59 g/l complete supplement mixture (CSM-ADE-HIS-LEU-TRP-URA, MP Biomedicals), 10 mg/ml adenine, 20 mg/ml uracil, 20 mg/ml histidine, 60 mg/ml leucine, 40 mg/ml tryptophan, 20 g/l agar) or synthetic media lacking the indicated amino acids or nucleobases. As sole carbon source, glucose (2%, 0.5%, 0.05%), galactose (2%), ethanol (3% / 0.1% glucose) or glycerol (3% / 0.1% glucose) was used.

### Generation of a *MAT* a / *MAT* α heterozygous double gene deletion collection

This study was conducted on the basis of results from the Kaeberlein laboratory that ranked yeast single gene knock-outs on the basis of their chronological lifespan [30]. From the 90 top scoring genes ranked, we crossed 82 *MAT* a with 85 *MAT* α most long-living mutants that were present in our version of the library (Additional file 1: Table S1) and used them as basis to create a heterozygous KO library. In order to select for diploids and to facilitate differential barcode sequencing, the *kanMX4* marker gene was replaced with *LEU2* in the 82 *MAT* a strains via homologous recombination (Additional file 2: Table S2). For this, the *LEU2* gene was amplified via PCR from plasmid pACT2 (Clontech, Table 1), the product was purified and transformed using the method described by Gietz et al. [33]. Positive transformants were selected on SC^-LEU^ and correct integration of the marker through homologous recombination was verified in single clones via PCR.

**Table 1:** **Plasmids used in the generation of mutant strains**

**Name**	**Source**	**Used for**
pACT2	Clontech	Cloning of *LEU2*
pAG25	Euroscarf	Cloning of *natMX4*
pAG32	Euroscarf	Cloning of *hphMX4*

The 82 marker-exchanged knock-out strains were then replicated into 9 × 96-well plates containing 50 μl YPD, whereas 85 *MAT* α knock-out strains were replicated into one 96-well plate containing 50 μl YPD and incubated at 30°C for 24 hours. 10 *MAT* α strains were combined each and then transferred into replicates of the *MAT* a plates. 9 plates with the *MAT* a / *MAT* α mixture were replicated on solid YPD media and incubated at 30°C for 2 days allowing mating to occur. Finally, diploids were selected on SC^-Leu^ + G_418_ (800 μg/ml) and subsequently pooled.

### Haploproficient screening and chronological ageing experiments

Competitive haploproficiency screens were conducted by inoculating 100 ml synthetic complete media (SC) + 2% glucose with overnight cultures of the wild type strain BY4743 and the library of heterozygous double mutants at an OD_600_ = 0.3 in a 1:1 ratio. Cultures were incubated at 30°C for 32 days on a shaking platform. Viability was determined every 2nd to 4th day by plating serial dilutions in triplicates onto SC media, allowing all cells to grow and onto SC^-Leu^, selecting for double mutants only. Colonies were counted and expressed as CFU/ml.

Non-competitive stationary phase survival experiments were conducted in quadruplicates by setting an overnight culture of the haploid strains to an OD_600_ = 0.3 in 100 ml SC media. Yeast was grown until stationary phase (2 days) and viability was recorded for 14 days.

### Isolation and sequencing of molecular barcodes from double heterozygous mutants

The two barcode sequences in the double mutants were amplified via PCR from genomic DNA preparations as described previously [34] and sequenced by Sanger’s didesoxynucleotide method [35]. The first barcode was amplified with the primers uptag/pFA6-rev (which binds within the *kanMX4* marker, Table 2), whereas the second barcode was amplified with the primers *LEU2*-fwd/downtag (which binds within the *LEU2* cassette, Table 2).

**Table 2:** **Primer sequences used in strain generation, verification, sequencing**

**Name**	**Used for**	**5′ - 3′ sequence**
kanMX4::*LEU2* - fwd	Replace genomic kanMX4 with *LEU2 (*from pACT2)	GCTGCAGGTCGACGGATCCCCGGGTTAAT TAAGGCGCGCCAGATCAACTGTGGGAATACTCAGGT
kanMX4::*LEU2* - rev	Replace genomic kanMX4 with *LEU2* (from pACT2)	TTTATTGTCAGTACTGATTAGAAAAACTCATCGAGCATC AAATGATCATGATTTTCTGTTACACC
*LEU2* – fwd	Downtag amplification and sequencing in double mutants	AGGTGTAACAGAAAATCATGAT
pFA6 – fwd	Downtag sequencing primer (*kanMX4*)	CTCGACATCATCTGCCCAGA
pFA6 – rev	Uptag sequencing primer (*kanMX4*)	GTCTGCAGCGAGGAGCCGTA
Uptag	Barcode forward primer	GATGTCCACGAGGTCTCT
Downtag	Barcode reverse primer	CGGTGTCGGTCTCGTAG
*Tal1*::HPH1-fwd	Replace genomic *TAL1* with hphMX4 (from pAG32)	TAGTAAAATACTTCTCGAACTCGTCACATATACGTGTACATAAGCTTGCCTTGTCCCCGCCG
Tal1::HPH1-rev	Replace genomic *TAL1* with hphMX4 (from pAG32)	GCATAAGGACATGGCCTAAATTAATATTTCCGAGATACTTCCTCGACACTGGATGGCGGCGT
NQM1::NTC-fwd	Replace genomic *NQM1* with natMX4 (from pAG25)	CCATCTAGAATGGGGTGGACAACATATAAAAGAAGAG
NQM1::NTC-rev	Replace genomic *NQM1* with natMX4 (from pAG25)	TACGTCAGAATTTTAATGAATATATAAGTCTGTACAC

### Yeast cultivation and extraction for metabolomics analysis

Sugar phosphate intermediates were extracted and quantified as described earlier [36]: Yeast strains were grown in synthetic complete media and were collected during logarithmic growth at an OD_600_ of 1.5 ± 0.05 using a rapid cold methanol quenching procedure [37]. Lysis of cells was achieved by FastPrep-24 cycles (MP Biomedicals, 3 × 20 s, 6.5 m/s) in organic extraction buffer (200 μl of 75:25 acetonitrile:water containing 0.2% formic acid). After a second extraction step with the same amount of water the supernatants of both steps were combined, dried (SpeedVac concentrator), resuspended in 100 μl water:acetonitrile (93:7) and then subjected to LC-MS/MS analysis. All solvents were used in HPLC grade or higher.

### LC-MS/MS measurements of sugar phosphates

1.5 μl of the extracted samples were injected on a C8 column (Agilent Zorbax SB-C8 Rapid Resolution HD, 2.1×100 mm, 1.8 μm) for separation with a gradient that started with isocratic flow at acetonitrile:water (12:88) for 3.5 min, a subsequent ramp to 38:62 acetonitrile:water for 2.5 min, an 30 sec washing step at 42% acetonitrile, followed by re-equilibration for 30 sec. This totaled in a cycle time of 7.5 min. 750 mg/l octylammonium acetate was added to all buffers as ion pairing reagent [38]. Analytes were quantified in MRM mode with an online coupled triple quadrupole mass spectrometer (Agilent 6460). Each metabolite was identified by similar retention time and fragmentation behavior and compared with externally measured commercially available standards. Optimal SRM transitions, ionization and fragmentation energies as well as ion source parameters can be found in [39]. Masshunter software (Agilent) was used for peak integration, external calibration (with repetitively measured standard dilution curves), absolute quantification and initial data analysis. All further data analysis was performed with R (http://www.r-project.org/).

### Determination of osmolality

Osmolality was determined by freezing point depression in a Knauer Osmometer (Knauer) from aliquots of 1 ml taken from the yeast cultures at the indicated time points throughout the chronological aging experiment. Yeast cultures were centrifuged twice at 16.000 × g for 2 min. 200 μl of the media were transferred to a vial and measured in triplicates.

### Stress tests

Stress tests addressing oxidative stress tolerance (with menadione and H_2_O_2_), salt and osmotic stress (NaCl, sorbitol) in liquid cultures or on solid plates were induced by adding the stressors to the given final concentration to the growth media as described previously [16].

### Quantitative RT-PCR

Overnight cultures were diluted to an OD_600_ = 0.15 and grown until mid-log phase (OD_600_ = 0.8 – 1.0) in SC + 2% glucose (for menadione, H_2_O_2_, NaCl, sorbitol treatment) and in SC with either 2% glucose, 2% galactose, 3% ethanol + 0.1% glucose or 3% glycerol + 0.1% glucose. In time course experiments (menadione, H_2_O_2_, NaCl, sorbitol treatment), samples were taken on the specified time points.

For glucose restriction experiments, yeast was pre-grown in triplicates until mid-log phase in 80 ml SC + 2% glucose media. Aliquots of 20 ml were centrifuged at 2000 × g for 2 min, and either frozen on dry-ice or transferred to media with low glucose concentration (0.5% or 0.05%) for 1 hr.

**Table 3:** **Primer sequences used in qRT-PCR**

**Gene product**	**Forward primer**	**Reverse primer**
*NQM1*	GAAGTATGAACCACAGGATT	CAAAATCTTATCCATGGCGT
*TAL1*	CCCATCATTGATCTTGGCTGC	GGCAGCTTGAATACCTTCCCA
*UBA4*	AAGAGAGCAGCTTGCCAAGA	TCCAGCACCAACTACCAAAA
*ATG27*	AGTTGTGCTTGGCGGAGTAT	CCTGCGAGAAGCATGATGTA
*TAF10*	CCAGGATCAGGTCTTCCGTA	TGCTGTCCTTGCAATAGCTG

Total yeast RNA was isolated using the RNAeasy Mini Kit (Qiagen). Two μg total yeast RNA was transcribed into cDNA using a 12–18 oligo dT primer and Moloney Murine Leukemia virus (M-MuLV) reverse transcriptase (NEB) according to the manufacturer’s instructions. qRT-PCR was performed on a Prism 7900HT cycler (Applied Biosystems) with gene-specific primers (Table 3) and Maxima SYBR Green/Rox qPCR Master Mix (Thermo Scientific). The relative expression ratio of the target genes was normalized to the geometric mean of the three endogenous reference genes (*UBA4, ATG27, TAF10*) following the method of Pfaffl et al. [40].

## Results

### A haploproficient interaction of the transaldolase *NQM1* and the transcription factor *VHR1* enriches cells at a late stage of stationary phase survival

**Figure 1:**
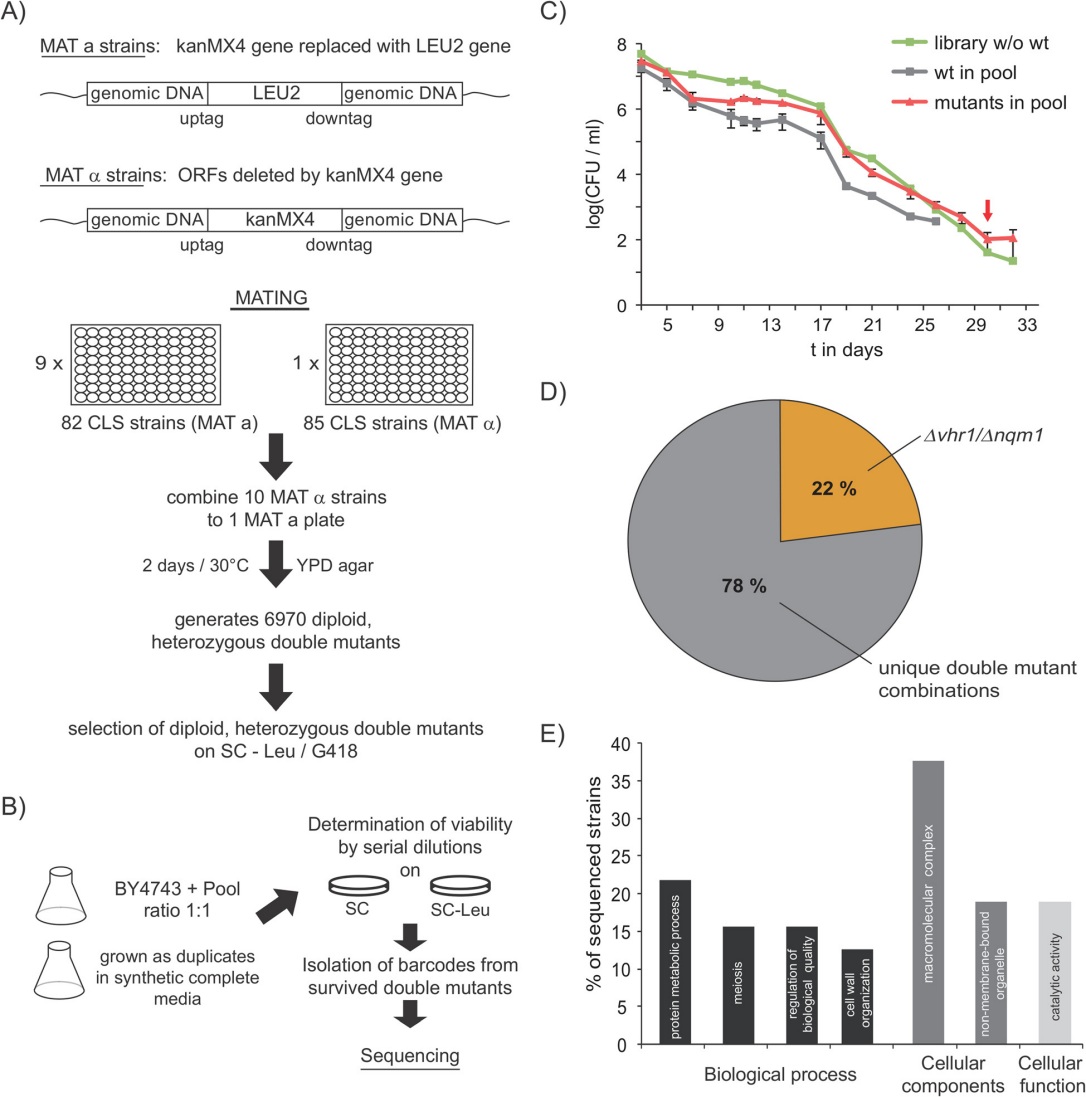
**Haploproficiency of a** ***VHR1/NQM1*** **double heterozygous mutant in late stationary phase. A)** Generation of a heterozygous haplotype library. The *kanMX4* marker present in the systematic knock-out strains was replaced by the *LEU2* in 82 *MAT* a strains, and these were cross-mated in 96-well plates with the corresponding *MAT α* collection. After 2 days mating on YPD, double mutants were selected on synthetic media lacking leucine and supplemented with G418. **B)** The double heterozygous mutant library was combined with the wild type strain BY4743 in a 1:1 ratio and grown in independent duplicates at 30°C for 32 days in synthetic complete media (SC). Viability was monitored every 2nd day by serial dilution plating **C)** Viability of cells during stationary phase in the double mutant library (green line), wild type within a competitive pool (grey line) or the mutants within the competitive pool (red line). The wild type lost viability in competitive pools at day 26, the double mutants at day 32. The arrow marks time point of clonal selection. These trends in linear scaling are illustrated in Additional file 3, Figure S7, the trends of separate pools in Additional file 4: Figure S8. **D)** Identification of surviving haplotypes. 78% of genotypes were unique, while the remaining cells were double-heterozygous for *Δvhr1* and *Δnqm1* **E)** Gene Ontology (GO) Term analyses of surviving double heterozygous yeast mutants on the basis of their single gene deletion.

Haploinsufficiency and haploproficient interactions have been described as a measure of genetic interactions that identifies a different kind of gene dependency as compared to synthetic genetic or physical interactions [41-46]. Haploproficiency and haploinsufficiency are of prime physiological importance in diploid (and polyploid) organisms, as randomly occurring mutations more likely result in the loss of function of just one instead of all copies of a gene [47]. In order to identify haploproficient interactions in the context of cellular ageing, we focused on a set of genes previously associated with chronological lifespan in budding yeast [30]. We took the corresponding 82 *MAT* a and 85 *MAT* α gene deletion strains as present in our copy of the yeast knock-out collection (Additional file 1: Table S1) and mated them with each other, adapting a multiple mating protocol established by Stelzl et al. [48]. The strategy in mating cells in repetitive batches of 10 strains facilitates a reasonable throughput, and avoids at the same time that a few strains possessing growth advantage can overgrow the library (Figure 1A).

In order to be able to select for diploids and to identify double heterozygous strains by means of barcode sequencing, we replaced the *kanMX4* gene in all *MAT* a strains by the *LEU2* marker (Figure 1A). After generation of the library containing up to 6970 *bona fide* dual heterozygous mutants, we inoculated this set of strains together with the diploid wild type BY4743*.* Viability of the population was followed for 32 days at 30°C by determining CFU/ml by spotting serial dilutions on synthetic complete (SC) and SC media lacking leucine (SC^-Leu^) to distinguish the double mutants from the wild type cells (Figure 1B). This measure was used as indicator whether the population contained haplotypes with increased stationary phase survival (Figure 1C, and Additional file 3: Figure S7 (linear scaling), Additional file 4: Figure S8, (for trends in separate pools)). We observed a strong decrease in viability during the first days of stationary phase growth; with day 3 (Figure 1C) the cells reached a plateau phase lasting until the 18th day. Then, cells started to lose viability rapidly and nearly linearly until the 30th day. At day 26, the wild type strain within the cultures had largely lost viability, but double mutants still produced viable colonies (Figure 1C). Thus, strains in the heterozygous library expressed prolonged stationary phase survival. From the surviving population, we randomly isolated 62 clones (31 from two parallel experiments). Via barcode sequencing [31], 39 haplotypes could be identified unambiguously which did lack two genes (Additional file 2: Table S2). 78% of the isolated strains possessed a private (unique) genotype (Figure 1D). These deleted genes were enriched for (overlapping) Gene ontology (GO) process terms *protein metabolic process, meiosis, regulation of biological activity, cell wall organization,* cellular components terms *macromolecular complex, non-membrane-bounded organelle* and GO function term *catalytic activity* (Figure 1E) when considering the deletant genes as background set (Additional file 1: Table S1). The remaining cells (22%) however corresponded to one sole enriched genotype. These strains were mainly isolated from one of the two experiments (Additional file 2: Table S2) and heterozygously deleted for the genes *VHR1* and *NQM1*, encoding for a transcription factor and an alternative transaldolase.

### *VHR1/NQM1* gene knock-outs possess increased clonogenicity at a late stage of stationary phase survival

**Figure 2:**
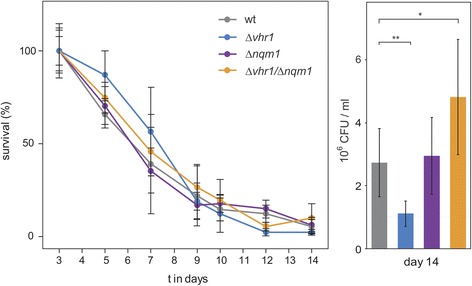
**Chronological ageing of haploid** ***Δvhr1*** **,** ***Δnqm1*** **and** ***Δvhr1/Δnqm1*** **cells.** Survival rates were determined in quadruplicate stationary cultures by spotting serial dilutions on synthetic complete media. Error bars +/− SD. Right panel: Number of colony forming cells on day 14. The values represent the amount of cells that are able to form colonies (clonogenicity) on fresh agar media. Error bars +/− SEM. Student’s t-test significance values p < 0.05 = *, p < 0.01 = **.

We then tested whether this haploproficiency translates into altered survival in stationary phase of haploid cells. We generated haploid *Δvhr1, Δnqm1* as well as their double mutant *Δvhr1/Δnqm1* strains and monitored their survival in stationary phase. Overall, all haploid strains had a shorter chronological lifespan compared to the diploid strains. This appears to be attributable to the *LEU2* marker, that had been repaired in the process of creating the diploid library [32]. The mortality during stationary phase, used to measure chronological lifespan, was similar among all mutant strains (Figure 2), and the initial clonogenicity on day three was similar among the strains (colony counts for wild type (51041667 CFUs/ml), *Δvhr1* (46083333 CFUs/ml), *Δnqm1* (48625000 CFUs/ml) and double mutant *Δvhr1/Δnqm1* (47708333 CFUs/ml). However, a significantly higher clonogenicity of the double mutant was detected at the last time point before stationary cultures lost viability (p < 0.05, Figure 2 right panel). *Vice versa*, a decreased clonogenicity was detected for the single *Δvhr1* mutant (p < 0.01). Thus, the haploproficient phenotype of the *vhr1/nqm1* double knock-out did correlate with a beneficial phenotype. Rather than a delayed decay in viability during the chronological ageing curve (known as chronological lifespan extension), the phenotype in both strains manifested as increased clonogenicity before stationary cultures lost survival.

### Altered concentration of glycolytic and PPP metabolites in *Δvhr1 and Δnqm1* strains and in their double mutants

**Figure 3:**
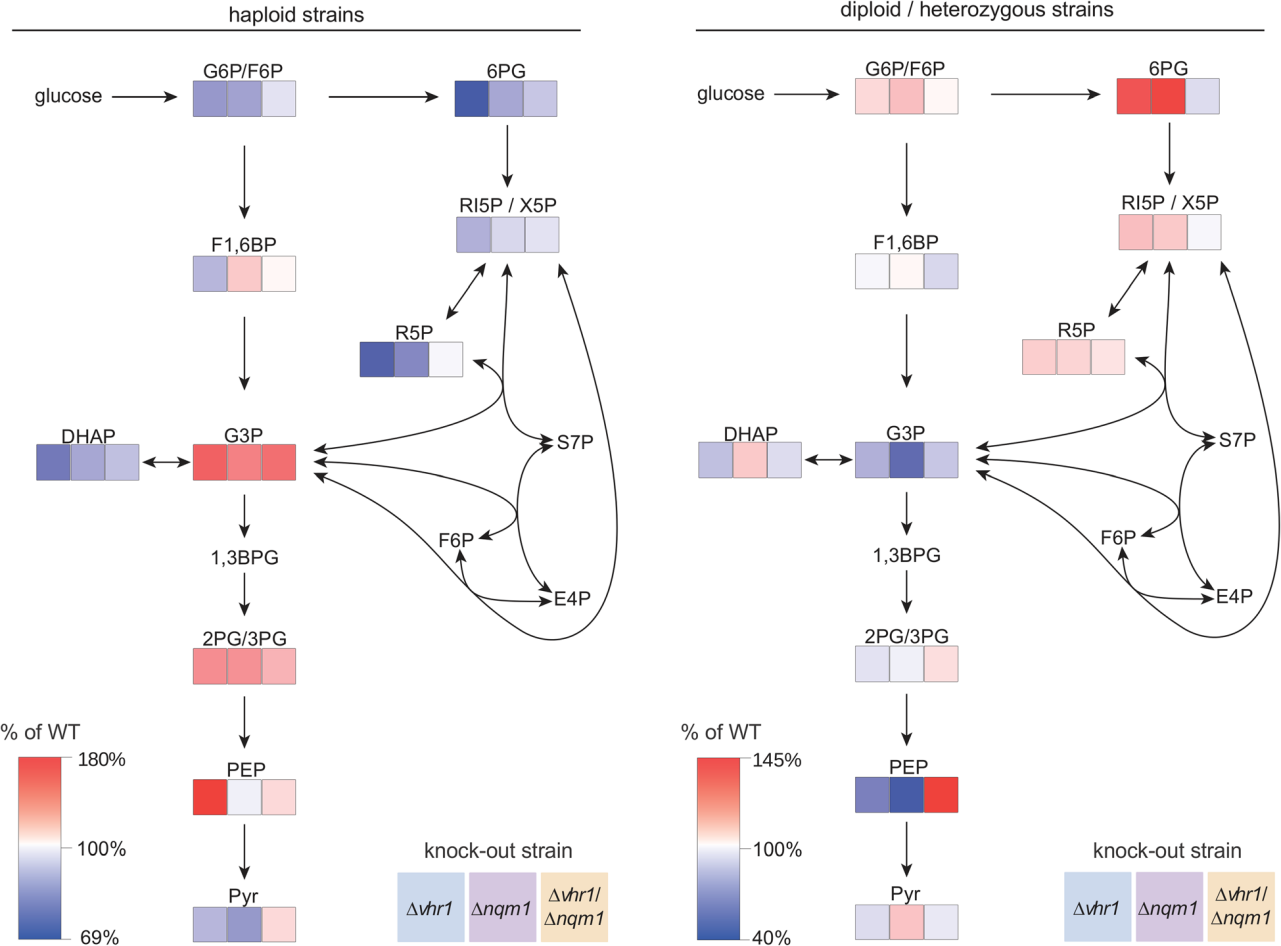
**Metabolic profiles of** ***Δvhr1, Δnqm1, Δvhr1/Δnqm1*** **deletion strains.** The sugar phosphate metabolic profile of haploid *Δvhr1, Δnqm1, Δvhr1/Δnqm1* deletion strains (left panel), and the corresponding diploid heterozygous mutants (right panel). Metabolite concentrations were normalized to wild type and color-coded. n = 3, G6P/F6P = glucose 6-phosphate/fructose 6-phosphate, F1,6BP = fructose 1,6-bisphosphate, G3P = glyceraldehyde 3-phosphate, DHAP = dihydroxyacetone phosphate, 1,3BPG = 1,3-bisphospoglycerate, 2PG/3PG = 2-phosphoglycerate/3-phosphoglycerate, PEP = phosphoenolpyruvate, Pyr = pyruvate, 6PG = 6-phosphogluconate, RI5P/X5P = ribulose 5-phosphate/xylulose-5-phosphate, R5P = ribose-5-phosphate, S7P = sedoheptulose 7-phosphate, E4P = erythrose 4-phosphate. The metabolites S7P, E4P, 1,3-BPG have not been quantified, and G6P/F6P, 2PG/3PG, RI5P/X5P have been quantified as sum.

*NQM1* is a paralogue of the transaldolase *TAL1*, an enzyme of the PPP [28]. We therefore tested whether *Δvhr1, Δnqm1* and the double knock-outs *Δvhr1/Δnqm1* (Figure 3 left panel) as well as the corresponding heterozygous strains (Figure 3 right panel) exhibited differences in PPP intermediate metabolite concentrations. Liquid chromatography tandem mass spectrometry (LC-MS/MS) was used to profile the concentrations of intermediate metabolites as described earlier [39]. Indeed, we identified altered concentration of glycolytic and PPP metabolites in the mutant strains. The haploid *Δvhr1* strain exhibited an up to 43% decreased concentration of metabolites of upper glycolysis (glucose 6-phosphate/fructose 6-phosphate (G6P/F6P), fructose 1,6-bisphosphate (F1,6BP), dihydroxyacetone phosphate (DHAP) and the oxidative and partially non-oxidative part of the pentose phosphate pathway (6-phosphogluconate (6PG), ribulose 5-phosphate/xylulose 5-phosphate (RI5P/X5P), ribose 5-phosphate (R5P)), and pyruvate (Pyr). In contrast, other metabolite levels of central and lower glycolysis (glyceraldehyde 3-phosphate (G3P), 2-phosphoglyerate/3-phosphoglycerate (2PG/3PG), phosphoenolpyruvate (PEP)) were elevated 65% above wild type level. The haploid *Δnqm1* strain displayed similar changes in metabolite levels as the *Δvhr1* mutant, but did not replicate the difference in PEP levels. The double mutant strain metabolic profile was less pronounced. Overall, its metabolite concentrations were more similar to the wild type strain (Figure 3).

Profiles of the heterozygous mutants were different to those of the haploid strains, but also here the metabolic profile of the double mutant was more similar to that of the wild type strain. Heterozygous deletion of *VHR1* or *NQM1* increased the concentration of PPP intermediates 6PG, RI5P/X5P and R5P, while *VHR1/Δvhr1 NQM1/Δnqm1* yeast instead displayed nearly wild type level concentrations of the PPP intermediates (Figure 3, right panel). This effect was also observed for metabolites of lower glycolysis, where the double mutant did not replicate the concentration changes of both single mutants. Hence, while both the *NQM1* and *VHR1* deletions affected the concentration of glycolytic and PPP intermediates, the double deletion reversed some of the effects, and yielded opposite concentration effects in other cases (Figure 3).

### *NQM1* is induced upon caloric restriction in a *VHR1* dependent manner

**Figure 4:**
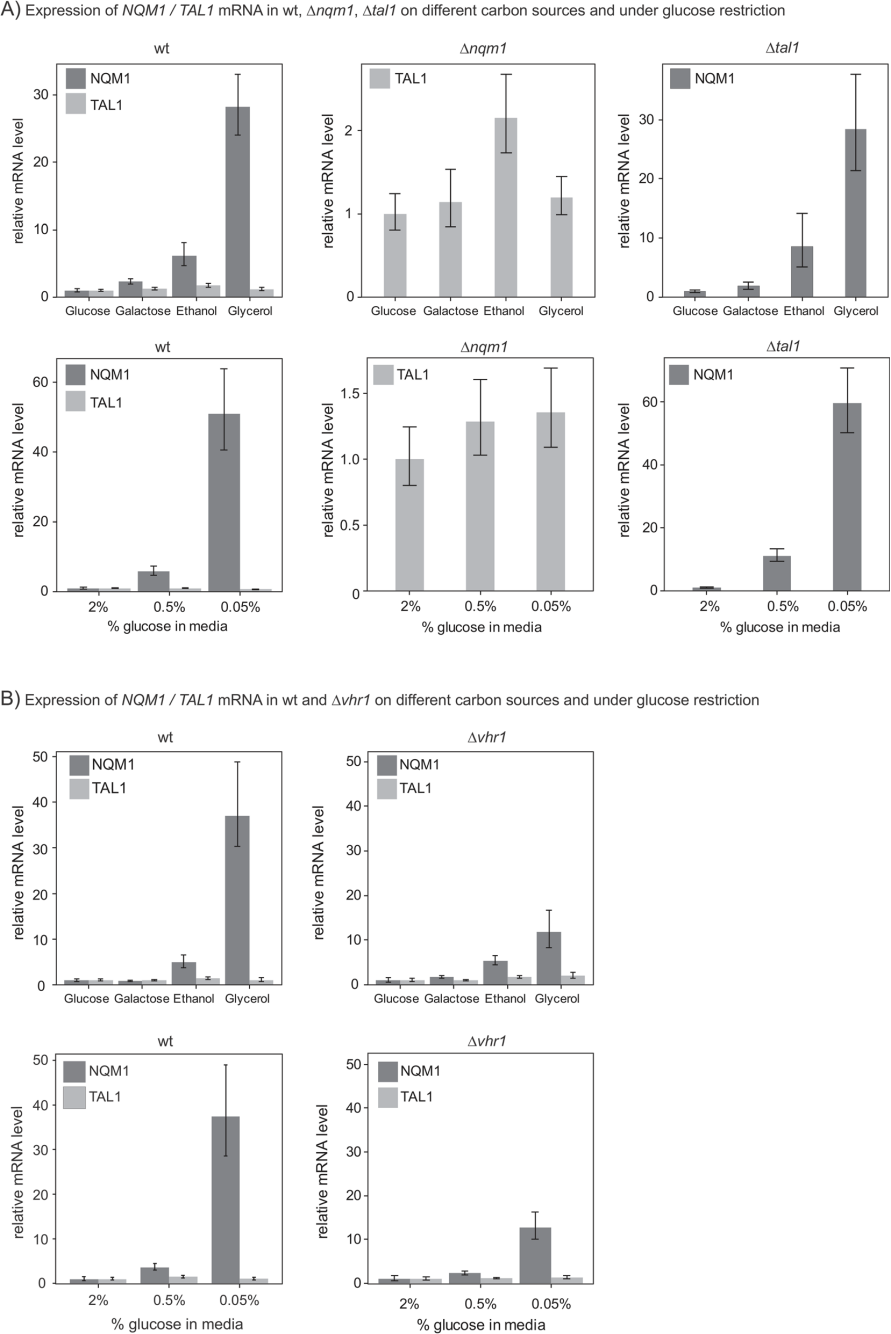
***NQM1*** **mRNA levels are induced by caloric restriction and on non-fermentable carbon sources. A)** Induction of *NQM1* on non-fermentable carbon sources and calorie restriction in wild type, *Δnqm1* and *Δtal1* deletion mutants. Expression of *NQM1* mRNA levels is induced on non-fermentable carbon sources (ethanol, glycerol) and on galactose, indicating that *NQM1* is subject to glucose repression. *TAL1* level instead are increased on ethanol only. **B)** Induction of *NQM1* on non-fermentable carbon sources and calorie restriction in wild type and *Δvhr1* mutant. *NQM1* induction is not suppressed by the deletion of *Δvhr1*, but strongly reduced. Samples for qRT-PCR have been generated in liquid media containing the indicated carbon source. Cells were harvested at mid-log phase (OD_600_ = 0.8–1.0). n = 3, Error bars, ± SD.

The PPP transaldolase Tal1p is a four-substrate enzyme, and required in the non - oxidative PPP to provide erythrose 4-phosphate and ribose 5-phoshate for the synthesis of amino acids and nucleotides, respectively [49,50]. While *in vitro NQM1* is active on the same substrates, under normal laboratory growth conditions Tal1p is fully sufficient to maintain PPP activity while deletion of its isozyme Nqm1p has no apparent phenotype. Hence, it was suggested that Nqm1p could be important under other growth conditions [51]. Interestingly, a classic metabolic profile of a transaldolase knockout [17] was not observed in our metabolomics experiments, which indicates that *NQM1* was not participating in the PPP, at least not in a comparable quantity as its paralogue *TAL1.* Thus, we investigated if mRNA expression of both transaldolase isozyme genes behaved similar upon changing nutrient supplementation by using qRT-PCR. For these experiments, wild type as well as *Δnqm1* and *Δtal1* yeast were grown in liquid media containing different carbon sources glucose, galactose, ethanol and glycerol. When cells were shifted towards the non-fermentable (gluconeogenetic) carbon sources ethanol or glycerol, *NQM1* mRNA levels increased about 30-fold (Figure 4A). *TAL1* expression levels instead did not change at the same level*. TAL1* expression increased only on ethanol, and around 5-fold only (Figure 4A, upper panel). Interestingly, the induction of *NQM1* was not affected by the deletion of *Δtal1,* and the level of *TAL1* not affected by the deletion *Δnqm1* (Figure 4A, middle and right panels)*.* Hence, non - fermentable carbon sources induce the expression of *NQM1*. The induction of *NQM1* was not influenced by presence or absence of its PPP isozyme *TAL1,* which in turn was not responsive to the carbon sources except a moderate induction on ethanol. As *TAL1* is a highly active enzyme, this indicates that the gene expression regulation of *NQM1* is not dependent on total transaldolase enzymatic activity.

The results instead implied that *NQM1* could be a glucose repressed gene. We tested the effects of glucose caloric restriction on its expression levels. Upon lowering the media glucose concentration from 2% over 0.5% to 0.05% mRNA levels of *NQM1* were induced up to 50-fold (Figure 4A, lower panels). In contrast, *TAL1* mRNA levels were not affected by glucose restriction, and a *Δtal1* did not influence *NQM1* expression. Thus, gene expression of paralogues is regulated independently from each other, and *NQM1* but not *TAL1*, is a glucose repressed gene.

As we had identified *NQM1* in concert with the transcription factor *VHR1* (Figure 1), we next addressed their genetic interplay upon the nutritional shift. We altered carbon sources and glucose concentration in the *Δvhr1* mutant and monitored *NQM1* levels in comparison to equally treated wild type cells. The absence of *VHR1* did not prevent the *NQM1* induction on non-fermentable carbon sources and upon glucose restriction. However, the expression levels of *NQM1* reached only 1/4th of the level compared to wild type cells (Figure 4B). Hence, *NQM1* is inducible on non - fermentable carbon sources and by glucose starvation. This function is independent to the canonical transaldolase *TAL1*, but requires the presence of the transcription factor *VHR1* for achieving wild-type expression levels.

### *NQM1* is induced upon osmotic stress

**Figure 5:**
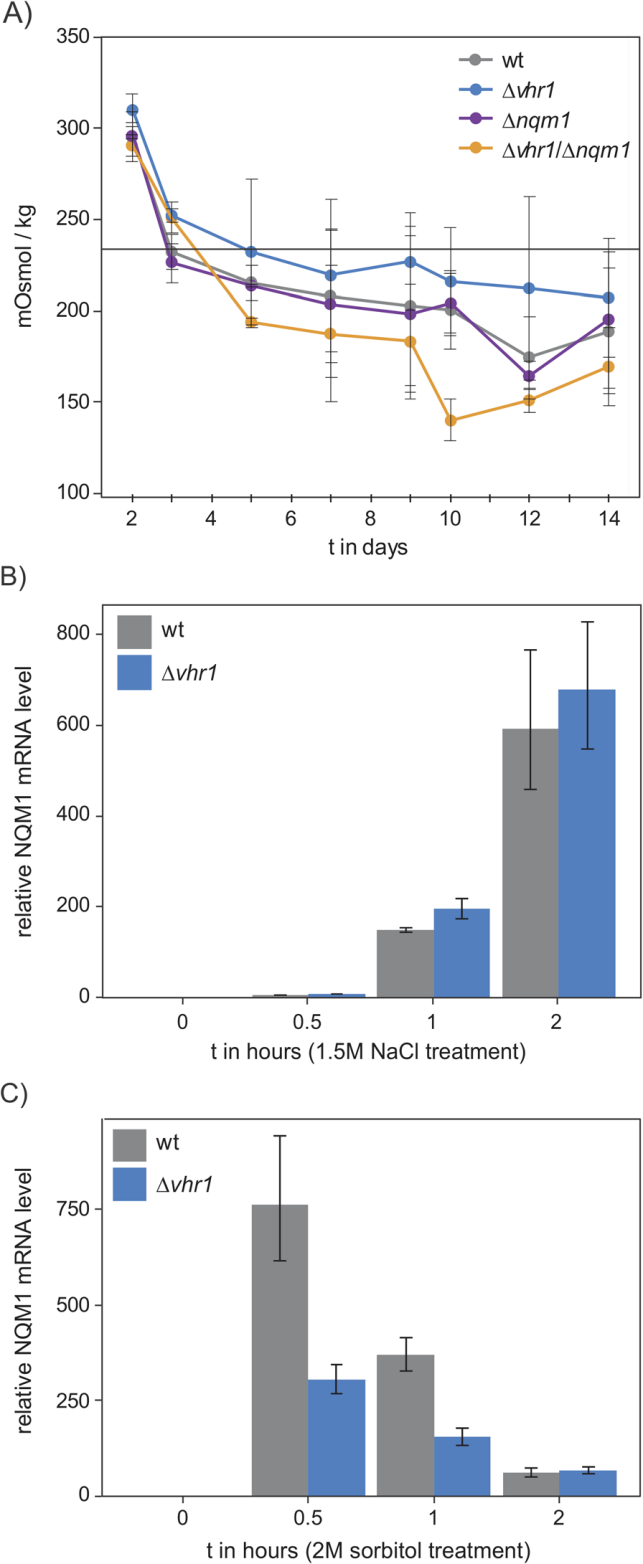
***NQM1*** **mRNA expression is dependent on osmolarity. A)** Osmolality was determined in triplicates by freezing-point depression. The grey bar at 233.75mOsmol/kg refers to the synthetic complete media osmolality prior to the experiment. All yeast strains depict a high osmolality at the start of the experiment which decreases during the chronological ageing below the control value (blank media) and remains stable until the end of the aging. Wild type (WT) and *Δnqm1* cells show a similar trend, whereas the decrease is stronger in the double mutant and diminished in *Δvhr1*. **B) + C)** mRNA expression of *NQM1* during the osmotic stress response in wild type and *Δvhr1* mutant yeast. Time courses of *NQM1* were generated by quantitative real-time-polymerase chain reaction (qRT-PCR) in wild type and *Δvhr1* yeast treated with NaCl or sorbitol for 0, 0.5, 1, and 2 hours. Upon NaCl treatment, *NQM1* was severely induced in both wild type and *Δvhr1* mutant yeast (Figure 5B). Upon sorbitol treatment, the induction pattern of *NQM1* was similar in wild type and *Δvhr1* mutant yeast, albeit the magnitude in the *Δvhr1* mutant was reduced (Figure 5C). All experiments were performed in triplicates. Samples for qRT-PCR have been generated in liquid media containing glucose (2%) as sole carbon source. NaCl, sorbitol was added to induce osmotic shock upon cells reached mid-log phase (OD_600_ = 0.8-1.0). Error bars, ± SD.

It has been reported that the anti-osmotic response of *HOG1-HOT1*, the major pathway of osmosis-controlled gene expression, affects the expression on *NQM1* [12]. Northern blot analysis has shown that *NQM1* is highly induced upon shift to NaCl in wild type cells, whereas a deletion in *HOG1* diminished *NQM1* expression more than 75% [12]. In order to test whether changes in osmotic potential are associated with the genetic interaction of *NQM1* and *VHR1*, we followed media osmolality during chronological aging. These experiments were conducted in stationary phase wild type cells as well as in the *Δvhr1, Δnqm1 and Δvhr1/Δnqm1* mutants. The osmolality of blank media was determined at 233.75 mOsmol/kg. Upon inoculating yeast, osmolality raised to 300–320 mOsmol/kg within 48 hrs indicating the release of yeast metabolites into the media. Afterwards, a slow but steady decline in osmolality until the end of the chronological lifespan was observed, which corresponds to a decline in media metabolite concentration. Osmolality decreased faster in the *Δvhr1/Δnqm1* double mutant, while in tendency slower in the *Δvhr1* mutant implicating metabolic differences between the strains (Figure 5A). Overall the osmolality did however not reach values which differed strongly to that of fresh yeast media. For this reason we consider it unlikely that difference in osmotic potential explains the different survival of *Δvhr1/Δnqm1* mutants. In agreement with this speculation, we did not detect altered resistance of *Δvhr1* and *Δnqm1* strains to NaCl, sorbitol, KCl or MnCl_2_ that induces changes in osmolality (data not shown).

However, we could confirm that osmotic stressors induce *NQM1* expression. We treated cells with NaCl and sorbitol, and consistent with the previous findings [12], *NQM1* mRNA level were dramatically induced up to 750-fold in the cells treated with NaCl and sorbitol (Figure 5B, C). The *Δvhr1* deletion did not significantly affect the *NQM1* gene expression on NaCl. On sorbitol however, a result analogous to glucose restriction was obtained: Deletion of *VHR1* did not prevent the induction of *NQM1*, but in the absence of this transcription factor, *NQM1* gene expression did not any longer reach wild type levels (Figure 5C).

### *NQM1* limits oxidant tolerance

**Figure 6:**
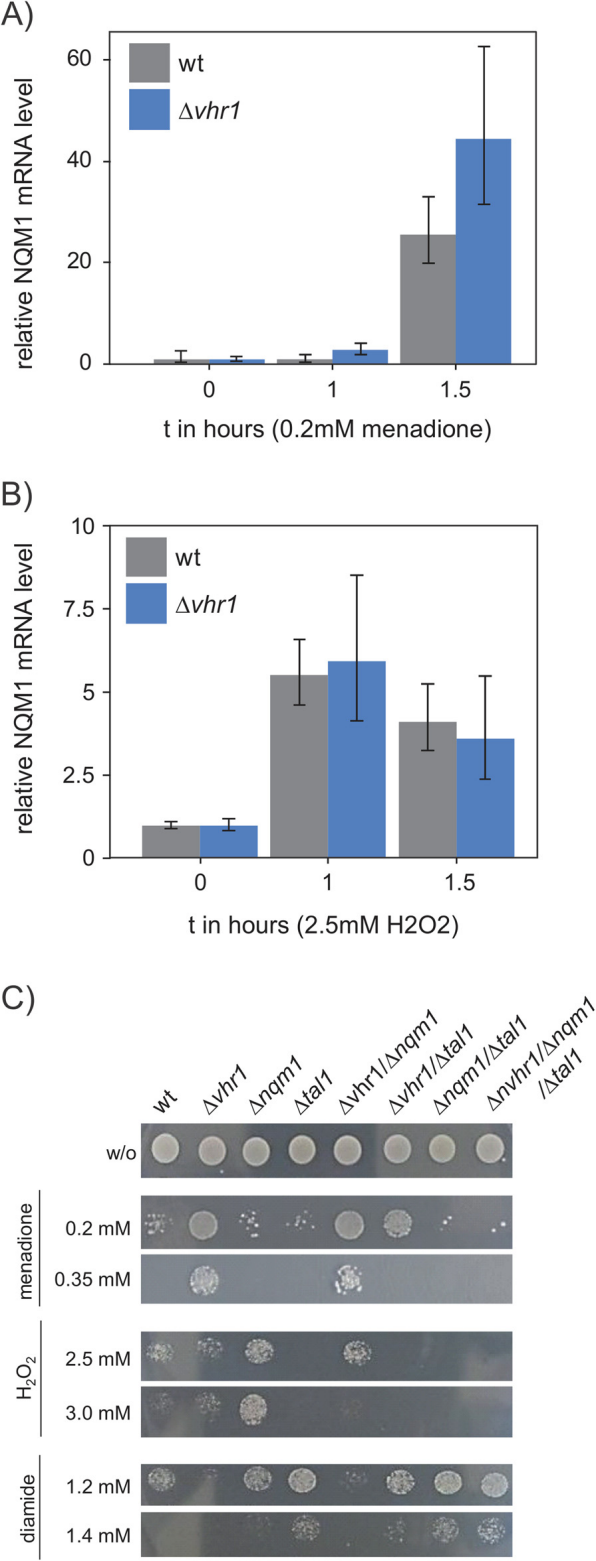
***NQM1*** **Expression and growth phenotypes of** ***NQM1/VHR1*** **mutants under oxidative stress. A)** *NQM1* mRNA expression in response to treatment with the oxidant menadione. Wild type and *Δvhr1* mutant yeast induced *NQM1* expression over time. **B)** H_2_O_2_ induces *NQM1* in both wild type and *Δvhr1* mutant; qRT-PCR was conducted on cells grown in liquid media containing glucose (2%) as sole carbon source. Oxidants were added to induce oxidative stress at mid-log growth phase (OD_600_ = 0.8–1.0). **C)** Oxidant tolerance spot tests on media containing different concentrations of the oxidant menadione, H_2_O_2_ and diamide. *Δvhr1* and *Δvhr1*/*Δnqm1* mutants exhibit increased tolerance to menadione, *Δnqm1* to H_2_O_2_, whereas the double mutants *Δvhr1*/*Δtal1*, *Δnqm1*/*Δtal1* and the triple mutant *Δvhr1*/*Δnqm1*/*Δtal1* were sensitive to this oxidant. *Δvhr1* and *Δvhr1*/*Δnqm1* mutants were sensitive to diamide, while all *Δtal1* mutants were resistant to this oxidant.

Oxidative stress is a hallmark of ageing [52-56]. As cellular oxidant tolerance a direct consequence of their metabolic activity [16] we continued by testing for expression changes of *NQM1* in cells treated with different concentrations of the oxidants menadione and hydrogen peroxide (H_2_O_2_). *NQM1* was strongly induced by menadione (25 - fold in wild type) and hydrogen peroxide (5.5 - fold in wild type). Deletion of *VHR1* did not prevent the induction of *NQM1* (Figure 6A, B). However, we detected higher expression of *NQM1* in the *Δvhr1* strain treated with menadione (Figure 6A).

Then, we tested whether cells deficient in *VHR1*, *NQM1* or *TAL1* would exhibit altered resistance to oxidants or reductants. Stress resistance was assayed by serial dilution spot testing for survival of a set of single, double- and triple deletion mutants comprising the genes *VHR1*, *NQM1*, *TAL1* on the oxidants diamide, H_2_O_2_, menadione, t-butyl hydroperoxide, cumene hydroperoxide and the reductants N-acetyl-cysteine, DTT, reduced glutathione. These experiments were conducted on media containing glucose, galactose, ethanol or glycerol. On glucose media, we identified a different tolerance of *NQM1* and *VHR1* mutants to menadione, H_2_O_2_ and diamide (Figure 6C): *Δvhr1* and *Δvhr1/Δnqm1* cells were resistant to menadione, *Δnqm1* to H_2_O_2_, whereas the *Δvhr1/Δtal1* mutant, the double transaldolase knock-out *Δnqm1/Δtal1* and in particular the triple knock-out strain *Δvhr1/Δnqm1/Δtal1* were sensitive to H_2_O_2_ (Figure 6C). The response to the thiol-oxidizing substance diamide differed to peroxide stresses. *Δvhr1* and *Δvhr1/Δnqm1* yeast were sensitive to diamide, while all *TAL1* mutants, *Δtal1, Δnqm1/Δtal1* and *Δvhr1/Δnqm1/Δtal1* mutants were resistant. Stress tests with the corresponding diploid, heterozygous strains did not detect difference to the wild type (Additional file 5: Figure S9). Taken together, *NQM1* is induced by oxidative stress in a partially *VHR1* dependent manner. *NQM1* and *VHR1* mutants exhibit a series of stress resistance and sensitivity phenotypes. This indicates that the interplay of the two genes is required during a native anti-oxidant stress response.

## Discussion

In this study we describe a competitive haploproficiency screening to identify genetic interactions that occur during yeast chronological ageing. We identified increased clonogenicity in late stationary phase of yeast mutants heterozygously deleted for *NQM1* and *VHR1*, encoding for the yeast transaldolase paralogue (*NQM1*) and a transcription factor (*VHR1*). Since a competition experiment among ~ 7000 double heterozygous deletion mutants reflects a complex system, we are limited to speculate about the dynamics that occurred during the course of the 30 day-long lasting stationary experiment. Previously, several studies showed that multiple factors like apoptosis [57], acetic acid accumulation [58] can affect the survival of yeast upon chronological aging. The latter could play a role in the selection dynamics of heterozygous mutants. Acetic acid has been proposed to be a major regulator of chronological lifespan [58] and has been linked to nutrient [59] and Ras signaling [60]. This advantage of the heterozygous double mutant was however also confirmed in individual, haploid mutants. Also in these experiments, the phenotype did express as higher clonogenicity of the double mutant culture at late time-points, right before the stationary cultures lost viability. The phenotype did however not express as decreased mortality upon yeast entering stationary phase. It is thus possible that the double mutant maintained longer viability in stationary phase by being more efficient in adaptive re-growth [57]. We are thus not considering this phenotype as an increase in chronological lifespan in the canonical sense, but refer to it as increased survival at late stationary phase. Being a different phenotype to a chronological lifespan extension, this phenotype could however cover an alternative feature of cellular survival; the ability to persist nutrient limiting conditions is an essential feature in the natural environment, and can provide significant population advantages.

Recent studies suggested that *NQM1* has either a direct role in gluconeogenic metabolism or in the supply of pentose phosphate precursors required for amino acid or nucleic acid biosynthesis under conditions under which regular synthesis from glucose 6-phosphate is difficult or insufficient [51]. Supporting parts of this assumption, we observed the induction of *NQM1* on different carbon sources, caloric restriction and several stress conditions. *NQM1* expression dominated over that of *TAL1* on non-fermentable carbon sources (ethanol/glycerol), in the presence of osmotic stressors, and under caloric restriction (low glucose) conditions (the latter indicating that *NQM1* is a glucose-repressed gene). However, we would like to note that *NQM1* expression levels did not respond to the deletion of *TAL1,* and thus not to changes in total transaldolase and PPP activity. In addition, we did not find canonical transaldolase substrates to accumulate in *Δnqm1* cells, and the oxidant resistance profile of *Δtal1* and *Δnqm1* strains were substantially different. Taken together these results indicate that despite its *in vitro* activity resembling Tal1p, *NQM1* could have yet unknown physiological substrates, instead of participating in the PPP.

We found *NQM1* induced under various stress conditions. This finding is consistent with a previous study focusing on the cumene hydroperoxide response, that identified a specific role of *NQM1* in the cellular response to the oxidizing molecules [61] and that *NQM1* is induced in those conditions [7]. The induction of *NQM1* after release of glucose repression and in stress situations was in part dependent on the *VHR1* transcription factor (Figures 4 and 5)*.* The DNA binding motif of Vhr1p is well studied, and it has been shown that the transcription factor is able to recognize bZIP-like DNA motifs and Gcn4-like motifs [62]. We could however not detect such binding sites for the *NQM1* locus using the Yeastract database, implying that the role of *VHR1* in regulating *NQM1* is likely indirect. Thus, further studies are needed to clarify the mechanisms and interactions which allow *VHR1* to influence the gene expression level of *NQM1*.

Yeast growth does change the growth media composition. The availability of nutrients, carbon or nitrogen sources will decline with time, while other metabolites (the exometabolome) accumulate [63-66]. Yeast preferentially metabolizes glucose through anaerobic metabolism resulting in the production of ethanol and carbon dioxide [67]. Changes in the concentration of soluble nutrients do affect the osmotic potential of the media. We detected a general decline in the media osmolality during in stationary phase survival (Figure 5A). Indicating that these changes reflect metabolic activity, this change in osmolality differed between wild type cells and the *VHR1* and *VHR1/NQM1* double mutants (Figure 5A). The changes in osmolality, albeit they were only moderate and thus unlikely a cause of toxicity, could however be recognized by mitogen activated protein (MAP) kinase pathways [68], in yeast represented by the high osmolarity glycerol (Hog1/Hot1) pathway [69]. *NQM1* has previously been shown to be induced during osmotic shock and that this induction was dependent upon *Hog1/Hot1* [12]. Our data is consistent with this observation; we find *NQM1* strongly induced upon adding osmotic stressors (Figure 5B, C). Of note, the osmotic response that is triggered by Hog1p has been associated with increased lifespan by enhancing glycerol production from glycolytic intermediates [70]. The mechanisms how double heterozygosity of *NQM1* and *VHR1* influences late stationary phase survival could thus potentially dependent on the induction of *NQM1* through osmotic signaling.

## Conclusions

In summary, the data presented here shows that combined deficiency in *NQM1 and VHR1* confers haploproficiency and extends clonogenicity at a late stage of yeast in stationary phase. We find that *NQM1* is a glucose repressed and stress induced isoform of the pentose phosphate pathway transaldolase *TAL1,* but is independently regulated and appears to have a different biochemical function compared to its paralogue. We find *NQM1* to be induced under conditions of caloric restriction, osmotic stress and in the presence of some oxidative stressors, and that full induction of *NQM1* requires the transcription factor *VHR1*. The detailed molecular mechanism by which *NQM1* is implicated in stationary phase metabolism needs to be resolved in future studies. However, our data shows that *NQM1* affects glucose metabolic activity in yeast stationary phase, under oxidative stress, osmotic stress and caloric restriction, and is thus implicated in difference facettes of the ageing process.

## Additional files

Additional file 1: Table S1. Yeast strains used in the study. Additional file 2: Table S2. Double mutants enriched in the competitive chronological lifespan experiments. Additional file 3: Figure S7. Survival trend (%) of the competitive chronological aging experiment. Viability of cells during competitive chronological aging shown for the wild types within the competitive pools (blue line – wt in pool1; green line – wt in pool 2) and the double mutants within the pools (red line – pool 1; purple line – pool 2). Additional file 4: Figure S8. Trend of viability in competitive chronological aging. The non-competitive wild type (grey dotted line), the competitive double mutant library only (dark grey dotted line), the competitive pool 1 separated into wild type (red line) and mutants (orange line), the competitive pool 2 separated into wild type (green line) and mutants (blue line). The non-competitive wild type shows prolonged survival whereas upon competition against the double mutant library, the wild type lost viability prior to the mutants in both pools. Additional file 5: Figure S9. Stress tolerance of haploid and diploid, heterozygous deletion strains. **A)** Stress tolerance of stationary phase cultures. The haploid *Δvhr1* and the double mutant *Δvhr1/Δnqm1* show increased stress tolerance upon various oxidants on fermentable (glucose) and non-fermentable (ethanol) carbon sources. **B)** Stress tolerance of diploid, heterozygous deletion strains. The stress tolerance is comparable to the wild type strain.
