# Supplementary material for: A haploproficient interaction of the transaldolase paralogue NQM1 with the transcription factor VHR1 affects stationary phase survival and oxidative stress resistance
Source: BMC Genet. 2015 Feb 11;16:13. doi: 10.1186/s12863-015-0171-6 (PMC4331311; doi:10.1186/s12863-015-0171-6)
Supplement: Additional file 1: Table S1. — Yeast strains used in the study. [file 12863_2015_171_MOESM1_ESM.pdf]

Additional File 1 Yeast strains for the generation of double mutants

MAT a strains		*MAT* α strains	
ΔYBL042C	*ΔYHR209W*	*ΔYBL042C*	*ΔYHR142W*
ΔYBL102W	*ΔYIL056W*	*ΔYBL102W*	*ΔYHR158C*
ΔYBL103C	*ΔYIL094C*	*ΔYBL103C*	*ΔYHR167W*
ΔYBR019C	*ΔYIL156W*	*ΔYBR019C*	*ΔYHR195W*
ΔYBR030W	*ΔYJL129C*	*ΔYBR030W*	*ΔYHR204W*
ΔYBR077C	*ΔYJL165C*	*ΔYBR077C*	*ΔYHR209W*
ΔYBR248C	*ΔYJR066W*	*ΔYBR248C*	*ΔYIL056W*
ΔYCL025C	*ΔYKL001C*	*ΔYCL025C*	*ΔYIL094C*
ΔYCR037C	*ΔYKL015W*	*ΔYCR037C*	*ΔYIL156W*
ΔYDR019C	*ΔYKL027W*	*ΔYDL172C*	*ΔYJL165C*
ΔYDR221W	*ΔYKL046C*	*ΔYDL173W*	*ΔYJR066W*
ΔYDR262W	*ΔYKL068W*	*ΔYDR019C*	*ΔYKL001C*
ΔYDR490C	*ΔYKL081W*	*ΔYDR221W*	*ΔYKL015W*
ΔYDR515W	*ΔYKL175W*	*ΔYDR262W*	*ΔYKL027W*
ΔYEL014C	*ΔYKL216W*	*ΔYEL014C*	*ΔYKL046C*
ΔYER040W	*ΔYKR007W*	*ΔYER040W*	*ΔYKL068W*
ΔYER091C-A	*ΔYKR034W*	*ΔYER091C-A*	*ΔYKL081W*
ΔYER132C	*ΔYKR095W*	*ΔYER132C*	*ΔYKL175W*
ΔYFR041C	*ΔYLL007C*	*ΔYFR041C*	*ΔYKR007W*
ΔYGL033W	*ΔYLL013C*	*ΔYGL033W*	*ΔYKR034W*
ΔYGL083W	*ΔYLR152C*	*ΔYGL083W*	*ΔYKR095W*
ΔYGL139W	*ΔYLR428C*	*ΔYGL139W*	*ΔYLL007C*
ΔYGL195W	*ΔYLR455W*	*ΔYGL195W*	*ΔYLL013C*
ΔYGL197W	*ΔYML066C*	*ΔYGL197W*	*ΔYLR152C*
ΔYGL203C	*ΔYML121W*	*ΔYGL203C*	*ΔYLR428C*
ΔYGR027C	*ΔYNL014W*	*ΔYGR027C*	*ΔYLR455W*
ΔYGR043C	*ΔYNL016W*	*ΔYGR043C*	*ΔYML066C*
ΔYGR044C	*ΔYNL021W*	*ΔYGR044C*	*ΔYML121W*
ΔYGR045C	*ΔYNL142W*	*ΔYGR045C*	*ΔYNL014W*
ΔYGR057C	*ΔYOL116W*	*ΔYGR054W*	*ΔYNL016W*
ΔYGR153W	*ΔYOR179C*	*ΔYGR057C*	*ΔYNL021W*
ΔYGR163W	*ΔYPL042C*	*ΔYGR059W*	*ΔYNL142W*
ΔYHL034C	*ΔYPL133C*	*ΔYGR153W*	*ΔYOL116W*
ΔYHR003C	*ΔYPL182C*	*ΔYGR163W*	*ΔYOR179C*
ΔYHR111W	*ΔYPR021C*	*ΔYGR237C*	*ΔYPL042C*
ΔYHR124W	*ΔYPR031W*	*ΔYGR289C*	*ΔYPL133C*
ΔYHR125W	*ΔYPR138C*	*ΔYHL034C*	*ΔYPL182C*
ΔYHR126C	*ΔYPR158W*	*ΔYHR003C*	*ΔYPR021C*
ΔYHR139C		*ΔYHR064C*	*ΔYPR031W*
ΔYHR142W		*ΔYHR111W*	*ΔYPR138C*

ΔYHR158C		*ΔYHR124W*	*ΔYPR158W*
ΔYHR167W		*ΔYHR125W*	
ΔYHR195W		*ΔYHR126C*	
ΔYHR204W		*ΔYHR139C*	
