# Supplementary material for: A haploproficient interaction of the transaldolase paralogue NQM1 with the transcription factor VHR1 affects stationary phase survival and oxidative stress resistance
Source: BMC Genet. 2015 Feb 11;16:13. doi: 10.1186/s12863-015-0171-6 (PMC4331311; doi:10.1186/s12863-015-0171-6)
Supplement: Additional file 2: Table S2. — Double mutants enriched in the competitive chronological lifespan experiments. [file 12863_2015_171_MOESM2_ESM.pdf]

AdditionalTable 2 List of strains enriched in the competitive chronological aging

#	ORFs deleted in double mutants		representing the genes		batch
**1**	YJL129C	YHR204W	TRK1	MNL1	batch 1
**2**	YBL103C	YER040W	RTG3	GLN3	batch 2
**3**	YCR037C	YKL081W	PHO87	TEF4	batch 2
**4**	YDL133C-A	YHR158C	RPL41B	KEL1	batch 1
**5**	YDL133C-A	YGL139W	RPL41B	FLC3	batch 1
**6**	YDL133C-A	YOR179C	RPL41B	SYC1	batch 1
**7**	YDL133C-A	YER132C	RPL41B	PMD1	batch 2
**8**	YDL172C	YGL083W	ORF dubious	SCY1	batch 2
**9**	YER040W	YDR515W	GLN3	SLF1	batch 2
**10**	YGL083W	YDR515W	SCY1	SLF1	batch 1
**11**	YGR045C	YER040W	ORF dubious	GLN3	batch 1
**12**	YGR045C	YFR041C	ORF dubious	ERJ5	batch 1
**13**	YGR045C	YGR161C	ORF dubious	RTS3	batch 2
**14**	YGR045C	YJR066W	ORF dubious	TOR1	batch 2
**15**	YGR057C	YJL129C	LST7	TRK1	batch 1
**16**	YGR059W	YGR043C	SPR3	NQM1	batch 1
**17**	YGR059W	YGL139W	SPR3	FLC3	batch 1
**18**	YHL034C	YKR095W	SBP1	MLP1	batch 1
**19**	YHL034C	YHR142W	SBP1	CHS7	batch 2
**20**	YHR139C	YHR125W	SPS100	ORF dubious	batch 2
**21**	YHR167W	YGR044C	THP2	RME1	batch 1
**22**	YHR167W	YHR003C	THP2	TCD1	batch 2
**23**	YIL056W	YHR111W	VHR1	UBA4	batch 1
**24**	YIL056W	YHR111W	VHR1	UBA4	batch 1
**25**	YIL056W	YGR043C	VHR1	NQM1	batch 2
**26**	YIL056W	YGR043C	VHR1	NQM1	batch 2
**27**	YIL056W	YGR043C	VHR1	NQM1	batch 2
**28**	YIL056W	YGR043C	VHR1	NQM1	batch 2
**29**	YIL056W	YGR043C	VHR1	NQM1	batch 2
**30**	YIL056W	YGR043C	VHR1	NQM1	batch 2
**31**	YIL056W	YGR043C	VHR1	NQM1	batch 2
**32**	YIL056W	YGR043C	VHR1	NQM1	batch 2
**33**	YIL056W	YGR043C	VHR1	NQM1	batch 2
**34**	YIL156W	YHR125W	UBP7	ORF dubious	batch 2
**35**	YJL129C	YML121W	TRK1	GTR1	batch 2
**36**	YJR066W	YHR124W	TOR1	NDT80	batch 1
**37**	YKL081W	YHL034C	TEF4	SBP1	batch 1
**38**	YNL014W	YJL129C	HEF3	TRK1	batch 1
**39**	YNL014W	YPL182C	HEF3	ORF dubious	batch 1
**40**	YPL133C	YJR066W	RDS2	TOR1	batch 1
**41**	YPL133C	YHR158C	RDS2	KEL1	batch 2
