# Supplementary material for: A haploproficient interaction of the transaldolase paralogue NQM1 with the transcription factor VHR1 affects stationary phase survival and oxidative stress resistance
Source: BMC Genet. 2015 Feb 11;16:13. doi: 10.1186/s12863-015-0171-6 (PMC4331311; doi:10.1186/s12863-015-0171-6)
Supplement: Additional file 3: Figure S7. — Survival trend (%) of the competitive chronological aging experiment. Viability of cells during competitive chronological aging shown for the wild types within the competitive pools (blue line – wt in pool1; green line – wt in pool 2) and the double mutants within the pools (red line – pool 1; purple line – pool 2). [file 12863_2015_171_MOESM3_ESM.pdf]

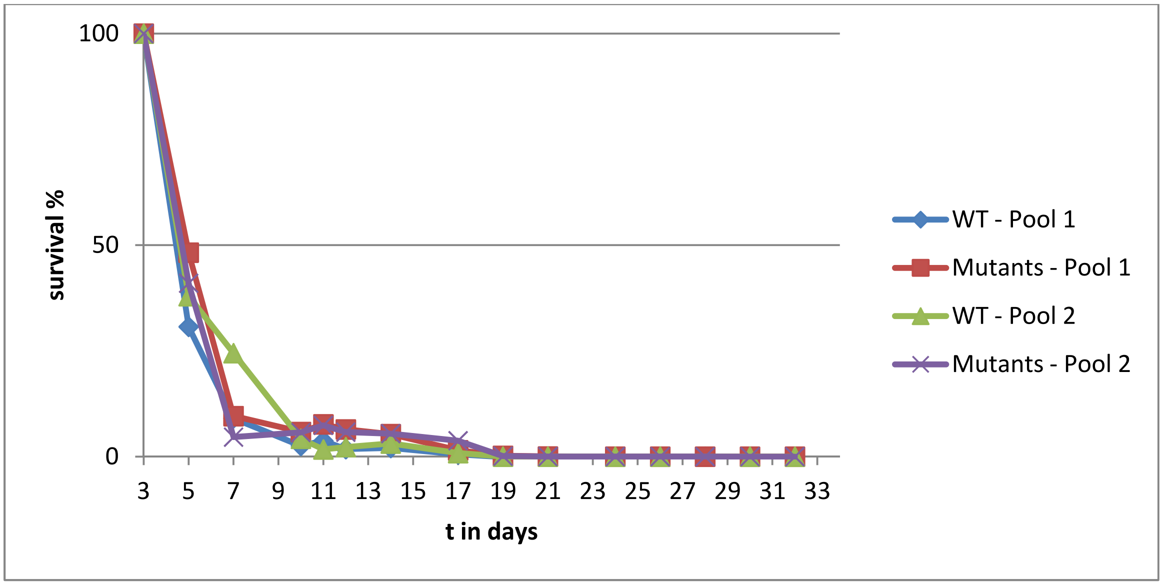

Figure 7: Survival trend (%) of the competitive chronological aging. Viability of cells during the competitive chronological aging experiment shown for the wild types within the competitive pools (blue line – wt in pool1; green line – wt in pool 2) and the double mutants within the pools (red line – pool 1; purple line – pool 2).
