# Supplementary material for: A haploproficient interaction of the transaldolase paralogue NQM1 with the transcription factor VHR1 affects stationary phase survival and oxidative stress resistance
Source: BMC Genet. 2015 Feb 11;16:13. doi: 10.1186/s12863-015-0171-6 (PMC4331311; doi:10.1186/s12863-015-0171-6)
Supplement: Additional file 4: Figure S8. — Trend of viability in competitive chronological aging. The non-competitive wild type (grey dotted line), the competitive double mutant library only (dark grey dotted line), the competitive pool 1 separated into wild type (red line) and mutants (orange line), the competitive pool 2 separated into wild type (green line) and mutants (blue line). The non-competitive wild type shows prolonged survival whereas upon competition against the double mutant library, the wild type lost viability prior to the mutants in both pools. [file 12863_2015_171_MOESM4_ESM.pdf]

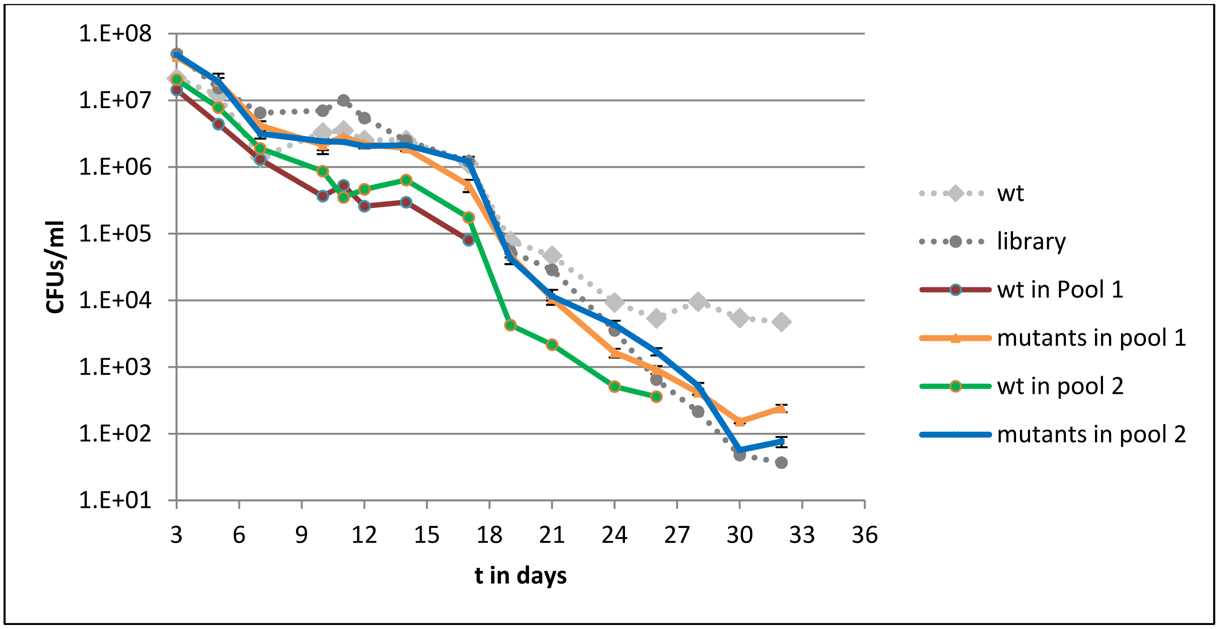

Figure 8: Trend of viability in competitive chronological aging. The non-competitive wild type (grey dotted line), the competitive double mutant library only (dark grey dotted line), the competitive pool 1 separated into wild type (red line) and mutants (orange line), the competitive pool 2 separated into wild type (green line) and mutants (blue line). The non-competitive wild type shows prolonged survival whereas upon competition against the double mutant library, the wild type lost viability prior to the mutants in both pools.
