# Supplementary material for: A haploproficient interaction of the transaldolase paralogue NQM1 with the transcription factor VHR1 affects stationary phase survival and oxidative stress resistance
Source: BMC Genet. 2015 Feb 11;16:13. doi: 10.1186/s12863-015-0171-6 (PMC4331311; doi:10.1186/s12863-015-0171-6)
Supplement: Additional file 5: Figure S9. — Stress tolerance of haploid and diploid, heterozygous deletion strains. A) Stress tolerance of stationary phase cultures. The haploid Δvhr1 and the double mutant Δvhr1/Δnqm1 show increased stress tolerance upon various oxidants on fermentable (glucose) and non-fermentable (ethanol) carbon sources. B) Stress tolerance of diploid, heterozygous deletion strains. The stress tolerance is comparable to the wild type strain. [file 12863_2015_171_MOESM5_ESM.pdf]

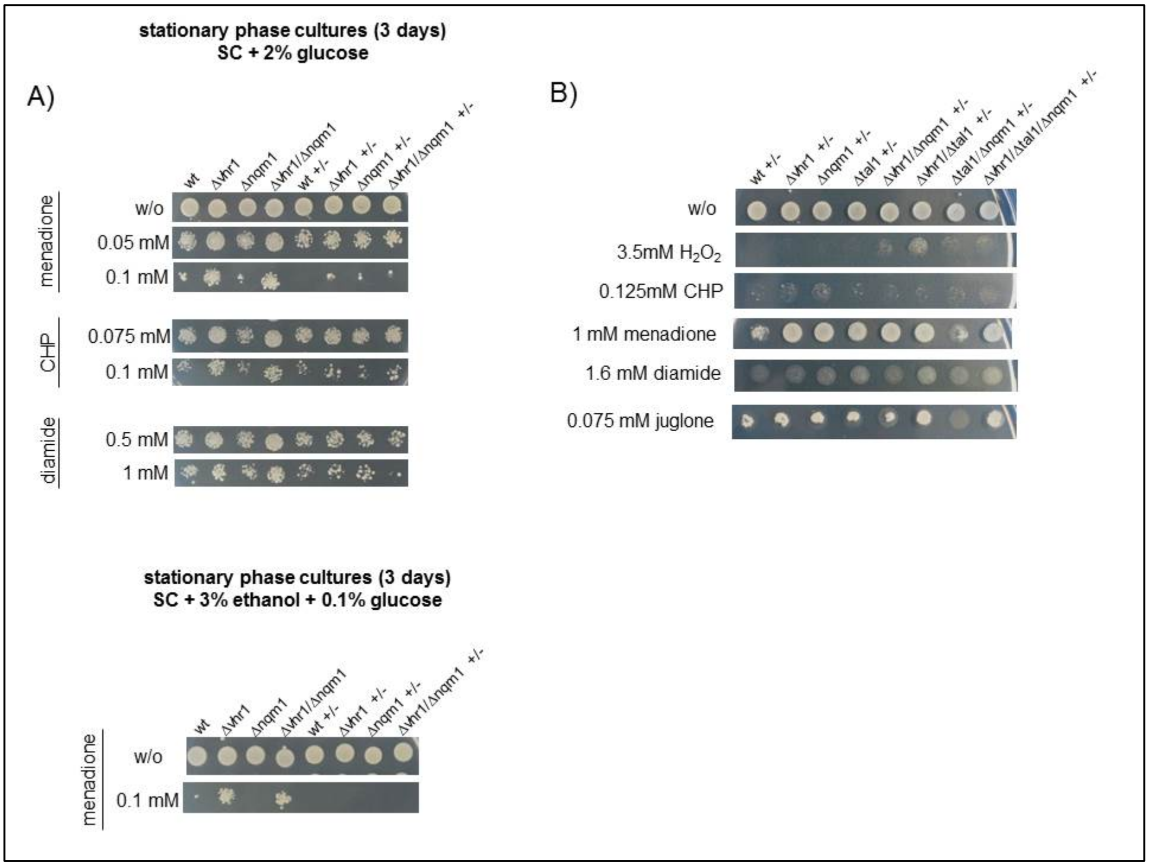

Figure 9 Stress tolerance of haploid and diploid, heterozygous deletion strains.

A) Stress tolerance of stationary phase cultures. The haploid *Δvhr1* and the double mutant *Δvhr1/Δnqm1* show increased stress tolerance upon various oxidants on fermentable (glucose) and non-fermentable (ethanol) carbon sources. **B)** Stress tolerance of diploid, heterozygous deletion strains. The stress tolerance is comparable to the wild type strain.
